# Diagnostic workup of cancer in patients with new-onset anaemia: a Danish cohort study in general practice

**DOI:** 10.1080/02813432.2021.1970934

**Published:** 2021-08-31

**Authors:** Astrid Boennelykke, Henry Jensen, Alina Zalounina Falborg, Lene Sofie Granfeldt Østgård, Anette Tarp Hansen, Kaj Sparle Christensen, Peter Vedsted

**Affiliations:** a Research Unit for General Practice, Aarhus C, Denmark; b Department of Public Health, Aarhus University, Aarhus C, Denmark; c Department of Haematology, Odense University Hospital, Odense C, Denmark; d Department of Clinical Epidemiology, Aarhus University Hospital, Palle Juul-Jensens, Aarhus N, Denmark; e Department of Clinical Biochemistry, Aalborg University Hospital, Aalborg, Denmark

**Keywords:** Anaemia, cohort studies, Denmark, early detection of cancer, general practice, primary health care

## Abstract

**Background:**

Anaemia is associated with adverse outcomes, including increased morbidity and all-cause mortality. Diagnostic workup of patients with anaemia is essential to detect underlying disease, especially undiagnosed malignancy.

**Objective:**

To describe the cancer-relevant diagnostic workup in patients with new-onset anaemia detected in general practice. An additional aim was to analyse associations between patient characteristics and the diagnostic workup.

**Design:**

Observational population-based cohort study using electronic laboratory and register data.

**Setting:**

Danish general practice.

**Subjects:**

Patients aged 40–90 years with new-onset anaemia (no anaemia in the preceding 15 months) detected in general practice. Patients were identified in Danish laboratory information systems and nationwide registries in 2014–2018.

**Main outcome measures:**

We measured the proportion of patients receiving predefined diagnostic investigations, that is, cancer patient pathway, colonoscopy, gastroscopy, computerised tomography (CT) scan, faecal test for haemoglobin, and bone marrow examination within three months of the anaemia index date.

**Results:**

We included 59,993 patients, and around half of the patients with ‘iron deficiency anaemia’, ‘anaemia of inflammation’, or ‘combined inflammatory iron deficiency anaemia’ had no cancer-relevant diagnostic investigations performed. Patients aged 60–79 years and patients with severe anaemia were more likely to have investigations performed, while patients with comorbidity were less likely to have investigations performed.

**Conclusion:**

Around half of the patients with anaemia subtypes that may indicate underlying cancer had no cancer-relevant diagnostic investigations performed. This may represent missed diagnostic opportunities. Future interventions are needed to improve the diagnostic workup of cancer in patients with anaemia, for example, laboratory alert systems and clinical decision support.

**KEY POINTS**
- The general practitioners are often the first to detect anaemia and its underlying disease (e.g. undiagnosed malignancy).
- Large-scale studies are needed on the diagnostic workup of patients with anaemia in general practice in relation to an underlying malignancy.
- This study shows that the majority of patients with anaemia had no cancer-relevant diagnostic investigations performed, which may cause diagnostic delay.
- Interventions seems needed to improve the diagnostic workup of cancer in these patients to ensure timely diagnosis.

## Introduction

Anaemia is a common condition, which is associated with frequent hospitalisation, increased morbidity, and higher all-cause mortality [1–3]. The most common subtypes of anaemia are iron deficiency anaemia (IDA) and anaemia of inflammation (AI), and these may coexist as combined inflammatory and iron deficiency anaemia (CIIDA) [4,5]. Anaemia can be caused by a variety of underlying diseases, including undiagnosed malignancy, which highlights the importance of determining the underlying cause [2,6].

In patients with unexplained IDA and CIIDA, diagnostic endoscopies are recommended to rule out gastrointestinal cancer [5,7,8]. The risk of gastrointestinal cancer has been reported to be 6%–10% in these patients, with domination of right-sided colorectal cancer with vague symptoms and signs, which underlines the importance of sufficient investigation [9,10]. The Danish diagnostic guidelines recommend referral of patients with unexplained AI to the cancer patient pathway for non-specific symptoms and signs (NSSC-CPP) [11]. The risk of cancer in patients with anaemia referred through a NSSC-CPP pathway has been reported to be 28% in patients with AI, 28% in patients with CIIDA, and 17% in patients with IDA [12]. Yet again, this stresses the importance to investigate for a potential undiagnosed malignancy in patients with anaemia.

General practitioners (GPs) are often the first to diagnose patients with anaemia. Thus, they have an essential role in assuring adequate workup of possible cancer in these patients. Previous studies have shown that patients in general practice with AI [13] or IDA [9,14,15] are not optimally investigated, which may cause diagnostic delay [16]. However, these were small-scale studies [9,13–15], and some were performed two decades ago [14,15]. Moreover, no previous studies have investigated the cancer-relevant diagnostic workup in patients with CIIDA or patients with unclassified anaemia.

We aimed to describe the cancer-relevant diagnostic workup in patients with new-onset anaemia detected in general practice. An additional aim was to analyse associations between patient characteristics and the diagnostic workup.

## Material and methods

We performed an observational population-based cohort study using data from Danish laboratory information systems [17] linked at an individual level to nationwide registries [18,19], using the unique civil registration number [20].

### Setting

This study is based on data from two of the five Danish healthcare regions, the Northern Denmark Region (0.6 million inhabitants) and the Central Denmark Region (1.3 million inhabitants) [21]. The Danish population (approx. 5.8 million inhabitants) has free access to a public tax-funded healthcare system [21]. Citizens need to consult their GP (99% are registered with a general practice) prior to hospital contact, except for emergencies, private practicing ophthalmologists and otorhinolaryngologists [22]. Thus, GPs act as gatekeepers to the specialised healthcare system.

All blood tests analysed at the departments of clinical biochemistry in the two regions registered in the laboratory information systems were included in this study [17]. Point-of-care tests analysed in general practice were not included.

### Study population

Patients aged 40–90 years living in the included regions were included in the study if registered with new-onset anaemia in the laboratory information systems. The anaemia was based on a blood test requested by a GP in the period from 1 April 2014 until 1 October 2018. Anaemia was defined as a haemoglobin level below 134 g/L (8.3 mmol/L) for men and below 118 g/L (7.3 mmol/L) for women according to the Danish reference intervals [23]. New-onset anaemia was defined as no anaemia in the laboratory information systems from general practice or a hospital in the 15 months preceding the date of anaemia (index date). Patients were not allowed to re-entry the cohort, and patients not listed with a general practice were excluded. Patients who moved in/out of the two regions or who died within the study period were censored in analyses.

### Exposure, outcome and covariates

#### Exposure

Patients with new-onset anaemia were categorised into anaemia subtypes based on blood tests requested by GPs within 31 days of the index date. We used the guideline for unexplained anaemia by the Danish Society for Gastroenterology and Hepatology [5,7]. The anaemia was categorised into IDA, CIIDA, AI or anaemia of ‘other causes’ [5,7]. The anaemia was categorised as unclassified if it could not be classified within these groups due to missing blood tests.

#### Outcome

Main outcome measures were diagnostic investigations, that is, cancer patient pathway (organ-specific or NSSC), colonoscopy, gastroscopy, computerised tomography (CT) scan of thorax, abdomen or pelvis, faecal test for haemoglobin (faecal occult blood test or faecal immunochemical test), and bone marrow examination. Diagnostic investigations were measured within three months from the index date. Further, the time to diagnostic investigations were measured during a six-month period.

Additionally, in the patients without any of the diagnostic investigations, we measured if they had other contacts instead. We included contacts to relevant hospital departments or to relevant private practicing specialists within three months from the index date. Hospital contacts were elective inpatient or outpatient visits.

Information on CPPs, colonoscopies, gastroscopies, CT scans, bone marrow examinations, and hospital contacts was obtained from the National Patient Register (NPR) [18]. Information on faecal tests was obtained from the laboratory information systems [17]. Information on contacts to private practicing specialists was obtained from the National Health Service Register (NHSR) [19].

#### Covariates

Covariates used were sex, age, educational level, disposable income, civil status, anaemia severity, and comorbidity. Information on sex and age was obtained from the Civil Registration System (CRS) [20]. Age was categorised as 40–49 years, 50–59 years, 60–69 years, 70–79 years, and 80–89 years. Information on educational level, disposable income, and civil status was retrieved from Statistics Denmark. Educational level was categorised as ‘low’ (ISCED levels 1 and 2), ‘medium’ (ISCED levels 3 and 4), and ‘high’ (ISCED levels 5 and 6) according to the International Standard Classification of Education (ISCED). Disposable income was divided into tertiles of ‘low’, ‘medium’, and ‘high’. Civil status was grouped into ‘living with a partner’ (married or registered partnership) and ‘living alone’. Information on anaemia severity was obtained from the laboratory information systems [17]. Anaemia severity was categorised into ‘mild’ (110 g/L-normal value), ‘moderate’ (80–110 g/L), and ‘severe’ (<80 g/L) according to the definitions by the World Health Organization (WHO) [24]. Information on comorbidity was retrieved from the Psychiatric Central Research Register (PCRR) and the National Patient Register (NPR) [18], and registered within 10 years prior to the anaemia index date. Comorbidity was categorised into 11 chronic disease groups (cardiovascular disease, hypertension, mental illness, diabetes, chronic obstructive pulmonary disease, neurological disorders, arthritis, inflammatory bowel disease, liver disease, kidney disease, and cancer), which have previously been used in research [25–27]. Number of comorbidities were classified into zero, one, two, and three or more.

### Statistical analysis

We calculated proportions of patients with diagnostic investigations and stratified by anaemia subtypes and sex. We calculated proportions of patients without any of the diagnostic investigations, but with contacts to hospital or to private practicing specialist, and stratified by anaemia subtypes. Adjusted proportions were estimated based on predictions at age 70–79 years following logistic regression analysis. In these analyses, we excluded patients who moved out of the included regions or died during the study period.

We calculated cumulative incidence proportions of patients with diagnostic investigations by applying the Aalen-Johansen estimator while considering death as competing risk. Patients were followed until the date of the first of the following events: diagnostic investigation, moving out, death, or end of follow-up. Analyses were stratified by anaemia subtypes and sex.

To investigate associations between patient characteristics and diagnostic workup, we estimated hazard ratios (HRs) by applying the Cox proportional hazard model. The proportional hazard assumption was evaluated from log-minus-log plots and assessed to be fulfilled. Patients were followed until the date of the first of the following events: any of the diagnostic investigations, moving out, death, or end of follow-up. Analyses were stratified by anaemia subtypes and adjusted for age group and sex.

Standard errors were modelled (in all analyses except for cumulative incidence proportions) to allow for intragroup correlations due to clusters of patients within general practice.

Analyses were performed with Stata^®^ version 16.

## Results

**Figure 1.**
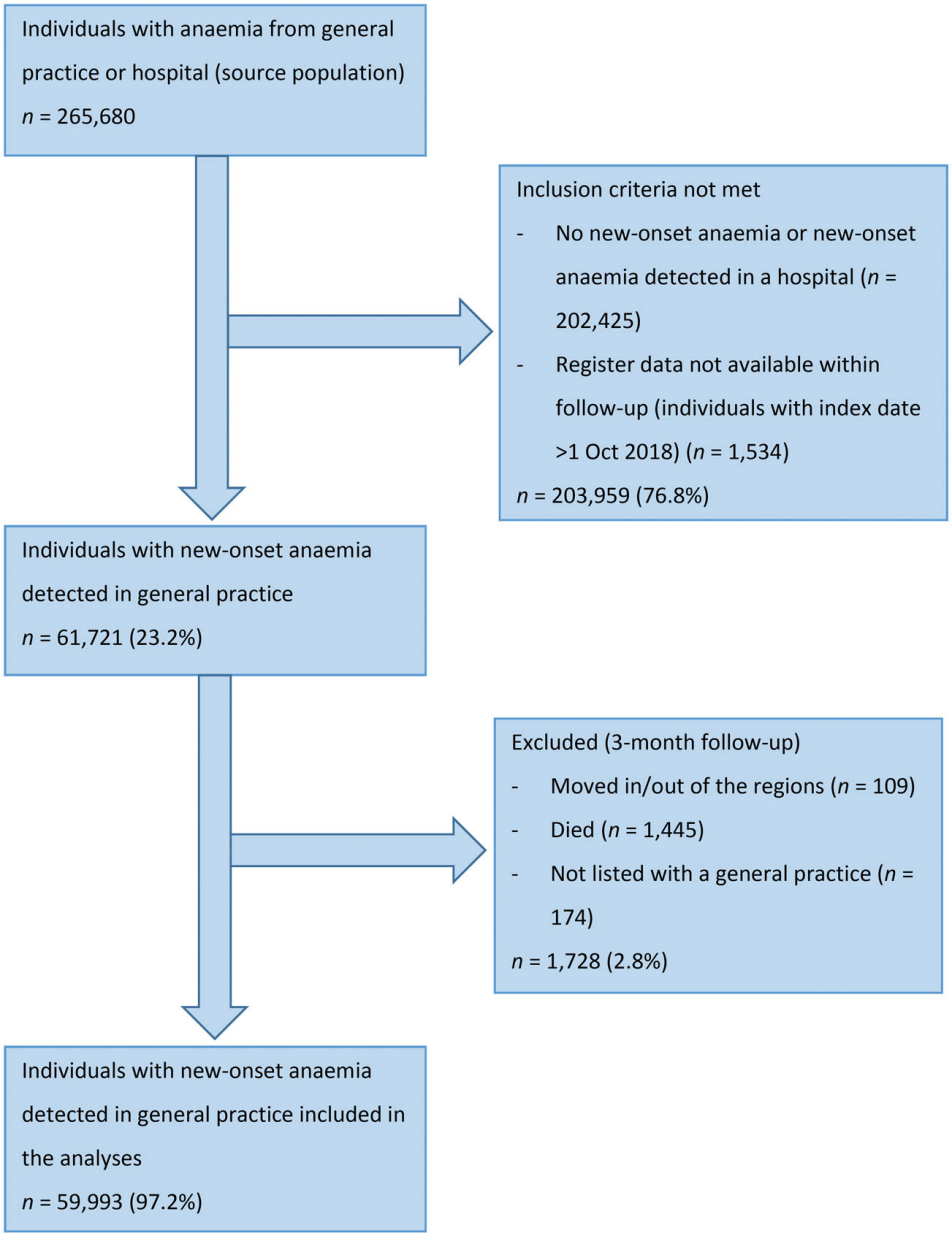
Flowchart of the study population.

Of the 59,993 included patients (Figure 1), 9,121 (15.2%) had IDA, 762 (1.3%) had CIIDA, 3,180 (5.3%) had AI, 5,650 (9.4%) had anaemia of ‘other causes’, and 41,280 (68.8%) had unclassified anaemia (Table 1). Patients with IDA were youngest (mean age 58.1 years) and patients with AI oldest (mean age 70.3 years). In total, 55.8% were men (ranging from 19.3% in IDA to 62.8% in unclassified anaemia), and 49.9% had no comorbidity (ranging from 44.8% in CIIDA to 64.6% in IDA) (Table 1).

**Table 1.** Patient characteristics of individuals aged 40–90 years with anaemia detected in general practice according to anaemia subtypes (*n* = 59,993).

Patient characteristics	IDA *n* (%)	CIIDA *n* (%)	AI *n* (%)	Other causes *n* (%)	Unclassified *n* (%)	Total *n* (%)
Total^a^	9,121 (15.2)	762 (1.3)	3,180 (5.3)	5,650 (9.4)	41,280 (68.8)	59,993 (100)
Age groups, years						
40–49	3,849 (42.2)	92 (12.1)	192 (6.0)	629 (11.1)	4,769 (11.6)	9,531 (15.9)
50–59	1,577 (17.3)	82 (10.8)	403 (12.7)	955 (16.9)	5,561 (13.5)	8,578 (14.3)
60–69	1,188 (13.0)	166 (21.8)	805 (25.3)	1,247 (22.1)	8,653 (21.0)	12,059 (20.1)
70–79	1,463 (16.0)	217 (28.5)	1,011 (31.8)	1,559 (27.6)	12,204 (29.6)	16,454 (27.4)
80–89	1,044 (11.4)	205 (26.9)	769 (24.2)	1,260 (22.3)	10,093 (24.5)	13,371 (22.3)
Sex						
Men	1,764 (19.3)	306 (40.2)	1,938 (60.9)	3,539 (62.6)	25,932 (62.8)	33,479 (55.8)
Women	7,357 (80.7)	456 (59.8)	1,242 (39.1)	2,111 (37.4)	15,348 (37.2)	26,514 (44.2)
Educational level						
Low	3,477 (38.1)	382 (50.1)	1,420 (44.7)	2,298 (40.7)	18,103 (43.9)	25,680 (42.8)
Medium	3,419 (37.5)	265 (34.8)	1,213 (38.1)	2,237 (39.6)	15,884 (38.5)	23,018 (38.4)
High	2,225 (24.4)	115 (15.1)	547 (17.2)	1,115 (19.7)	7,293 (17.7)	11,295 (18.8)
Income						
Low	2,396 (26.3)	288 (37.8)	1,162 (36.5)	1,894 (33.5)	14,226 (34.5)	19,966 (33.3)
Medium	2,821 (30.9)	270 (35.4)	1,046 (32.9)	1,777 (31.5)	13,930 (33.7)	19,844 (33.1)
High	3,904 (42.8)	204 (26.8)	972 (30.6)	1,979 (35.0)	13,124 (31.8)	20,183 (33.6)
Civil status						
Living with a partner	5,166 (56.6)	382 (50.1)	1,774 (55.8)	3,235 (57.3)	23,353 (56.6)	33,910 (56.5)
Living alone	3,955 (43.4)	380 (49.9)	1,406 (44.2)	2,415 (42.7)	17,927 (43.4)	26,083 (43.5)
Anaemia severity^b^						
Mild	3,871 (42.4)	534 (70.1)	2,419 (76.1)	4,796 (84.9)	35,736 (86.6)	47,356 (78.9)
Moderate	4,578 (50.2)	217 (28.5)	721 (22.7)	808 (14.3)	5,127 (12.4)	11,451 (19.1)
Severe	672 (7.4)	11 (1.4)	40 (1.3)	46 (0.8)	417 (1.0)	1,186 (2.0)
No. of comorbidities						
0	5,891 (64.6)	341 (44.8)	1,659 (52.2)	2,851 (50.5)	19,168 (46.4)	29,910 (49.9)
1	1,714 (18.8)	193 (25.3)	796 (25.0)	1,462 (25.9)	10,722 (26.0)	14,887 (24.8)
2	912 (10.0)	122 (16.0)	471 (14.8)	873 (15.5)	6,966 (16.9)	9,344 (15.6)
≥3	604 (6.6)	106 (13.9)	254 (8.0)	464 (8.2)	4,424 (10.7)	5,852 (9.8)
Type of comorbidity^c^						
Cardiovascular disease	1,492 (16.4)	201 (26.4)	757 (23.8)	1,425 (25.2)	11,572 (28.0)	15,447 (25.7)
Hypertension	1,383 (15.2)	185 (24.3)	649 (20.4)	1,229 (21.8)	9,988 (24.2)	13,434 (22.4)
Mental illness	737 (8.1)	77 (10.1)	218 (6.9)	457 (8.1)	3,601 (8.7)	5,090 (8.5)
Diabetes	716 (7.9)	91 (11.9)	215 (6.8)	479 (8.5)	4,636 (11.2)	6,137 (10.2)
COPD	401 (4.4)	93 (12.2)	218 (6.9)	277 (4.9)	2,585 (6.3)	3,574 (6.0)
Neurological disorder	171 (1.9)	26 (3.4)	82 (2.6)	187 (3.3)	1,325 (3.2)	1,791 (3.0)
Arthritis	58 (0.6)	7 (0.9)	40 (1.3)	58 (1.0)	428 (1.0)	591 (1.0)
IBD	84 (0.9)	14 (1.8)	22 (0.7)	56 (1.0)	417 (1.0)	593 (1.0)
Liver disease	97 (1.1)	21 (2.8)	52 (1.6)	71 (1.3)	523 (1.3)	764 (1.3)
Kidney disease	64 (0.7)	16 (2.1)	54 (1.7)	83 (1.5)	771 (1.9)	988 (1.6)
Cancer	355 (3.9)	60 (7.9)	272 (8.6)	417 (7.4)	3,442 (8.3)	4,546 (7.6)

AI: anaemia of inflammation; CIIDA: combined inflammatory iron deficiency anaemia; COPD: chronic obstructive pulmonary disease; IBD: inflammatory bowel disease; IDA: iron deficiency anaemia; No.: number; Unclassified: the anaemia is not classifiable according to a guideline.

^a^Total percentages are shown in the rows. Other variables are shown in the columns.

^b^Anaemia severity was categorised into: mild anaemia (110 g/L to normal), moderate anaemia (80–110 g/L), severe anaemia (<80 g/L) (according to WHÓs guidelines). Units were converted from g/L to mmol/L (110 g/*L* = 6.8 mmol/L, 80 g/*L* = 5 mmol/L).

^c^Type of comorbidity was categorised according to the chronic disease groups.

### Diagnostic investigations

In total, 73.7% (CI: 72.8–74.7) of men and 70.8% (CI: 69.7–71.9) of women had none of the diagnostic investigations performed. Around half of the patients with IDA, CIIDA, or AI had none of the diagnostic investigations performed (ranged from 45.4% in AI to 62.2% in IDA). Among women, the proportion ranged from 45.4% (CI: 42.3–48.6) in AI to 76.2% (CI: 75.2–77.3) in unclassified anaemia. A similar pattern was seen among men, although a lower proportion was seen among men with IDA (47.3%, CI: 44.4–50.2) compared to women with IDA (62.2%, CI: 60.3–64.0) (Figure 2).

Figure 2.Proportion (%)^a^ of patients with anaemia with diagnostic investigations within three months from the index date (stratified by anaemia subtypes and sex) (*n* = 59,993). AI: anaemia of inflammation; Bone marrow: bone marrow examination; CIIDA: combined inflammatory iron deficiency anaemia; CPP: cancer patient pathway; CT scan: computerised tomography scan; IDA: iron deficiency anaemia. ^a^Adjusted percentages were calculated by setting age at 70–79 years. Error bars = 95% Confidence intervals. Percentages add up to more than 100% as some patients had more than one of the investigations performed.

**Figure:**
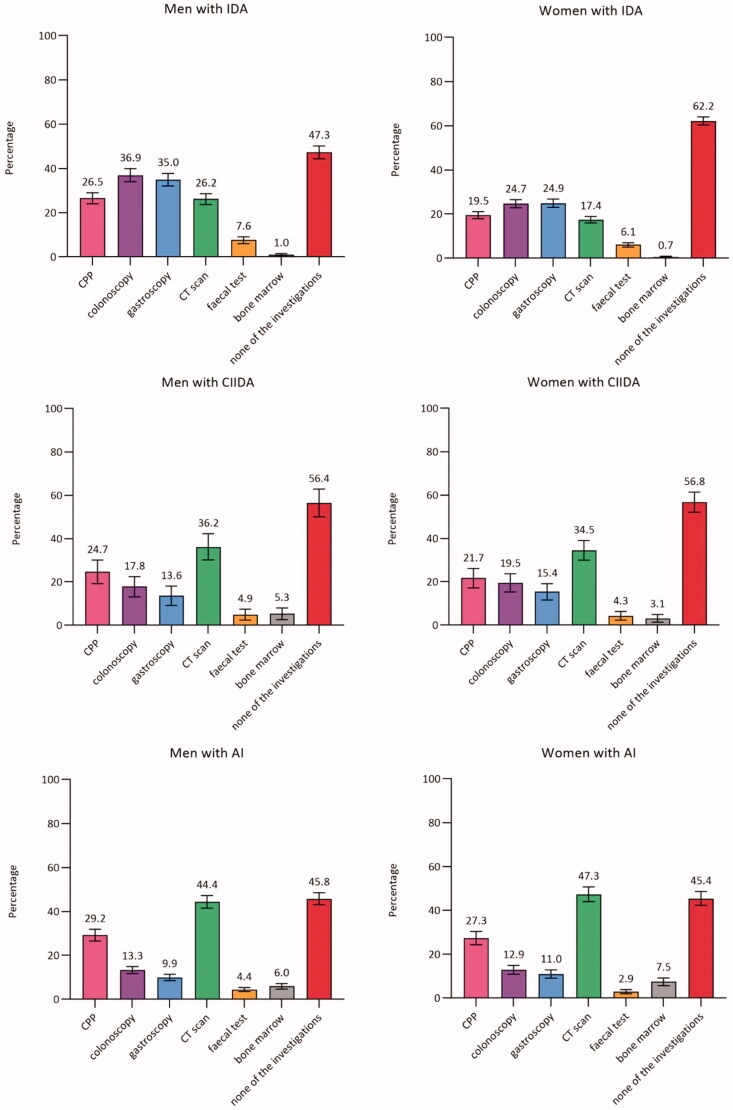

**Figure:**
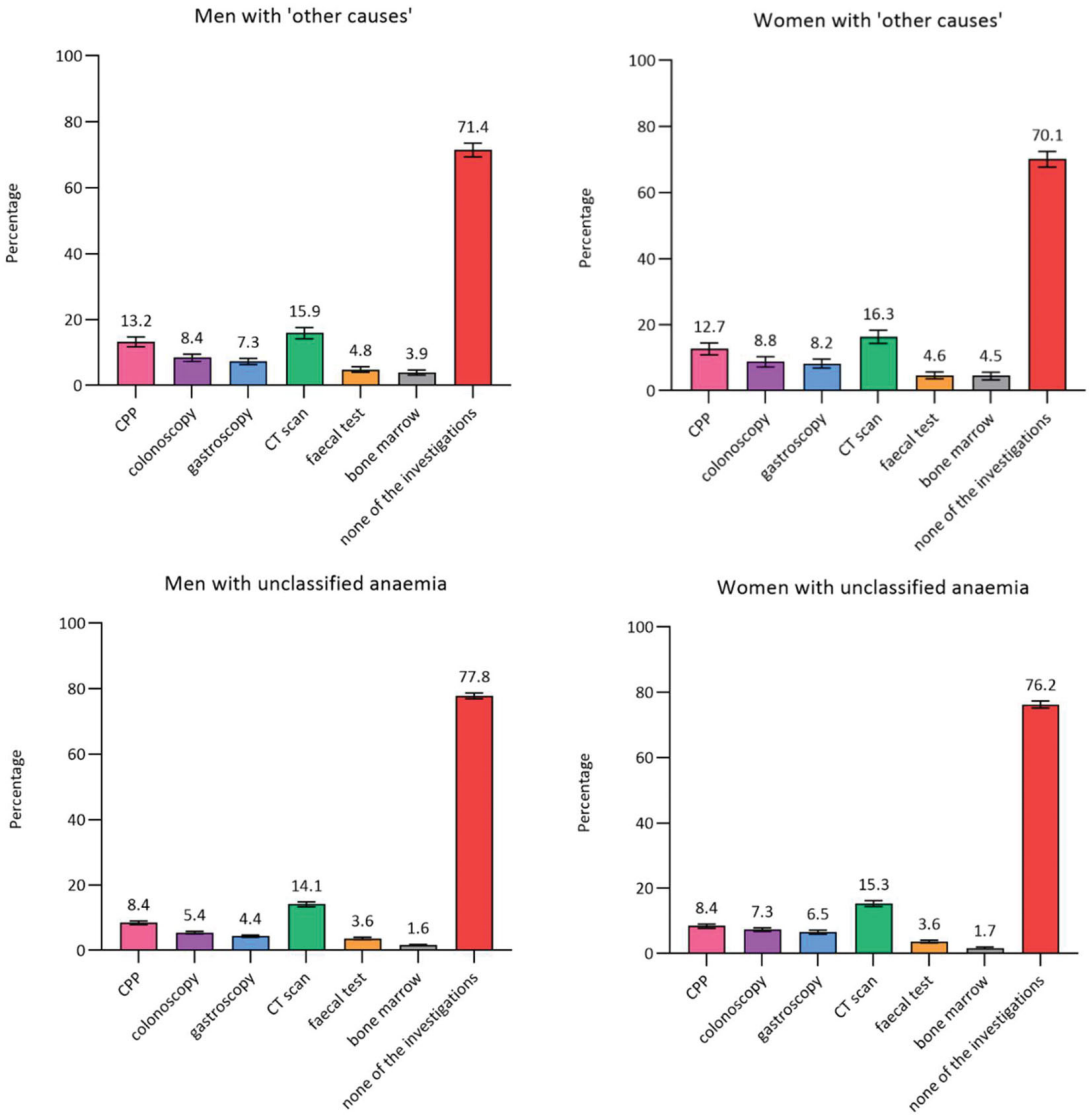

Moreover, we performed a sub analysis in women with IDA aged 50 to 90 years; and this analysis showed that 58.7% (CI: 56.7-60.7) did not have any of the diagnostic investigations performed. Additionally, we performed a sub analysis in patients with moderate or severe anaemia; and this analysis showed that a range from 26.4% (CI: 15.0-37.9) in men with CIIDA to 62.5% (CI: 60.1-65.0) in women with unclassified anaemia did not have any of the diagnostic investigations performed. For IDA, the most used investigation was colonoscopy in men (36.9%, CI: 34.0–39.9) and gastroscopy in women (24.9%, CI: 23.0–26.8). For all other anaemia subtypes, the most used investigation was CT scan (ranging from 14.1% (CI: 13.3–14.8) in men with unclassified anaemia to 47.3% (CI: 44.0–50.7) in women with AI).

Across all anaemia subtypes, the majority of investigations were performed during the first three months after the index date (Figure 3).

Figure 3.Cumulative incidence proportion of patients with anaemia with diagnostic investigations during a six-month follow-up period (stratified by anaemia subtypes and sex) (*n* = 59,463). AI: anaemia of inflammation; Bone marrow: bone marrow examination; CIIDA: combined inflammatory iron deficiency anaemia; CPP: cancer patient pathway; CT scan: computerised tomography scan; IDA: iron deficiency anaemia.

**Figure:**
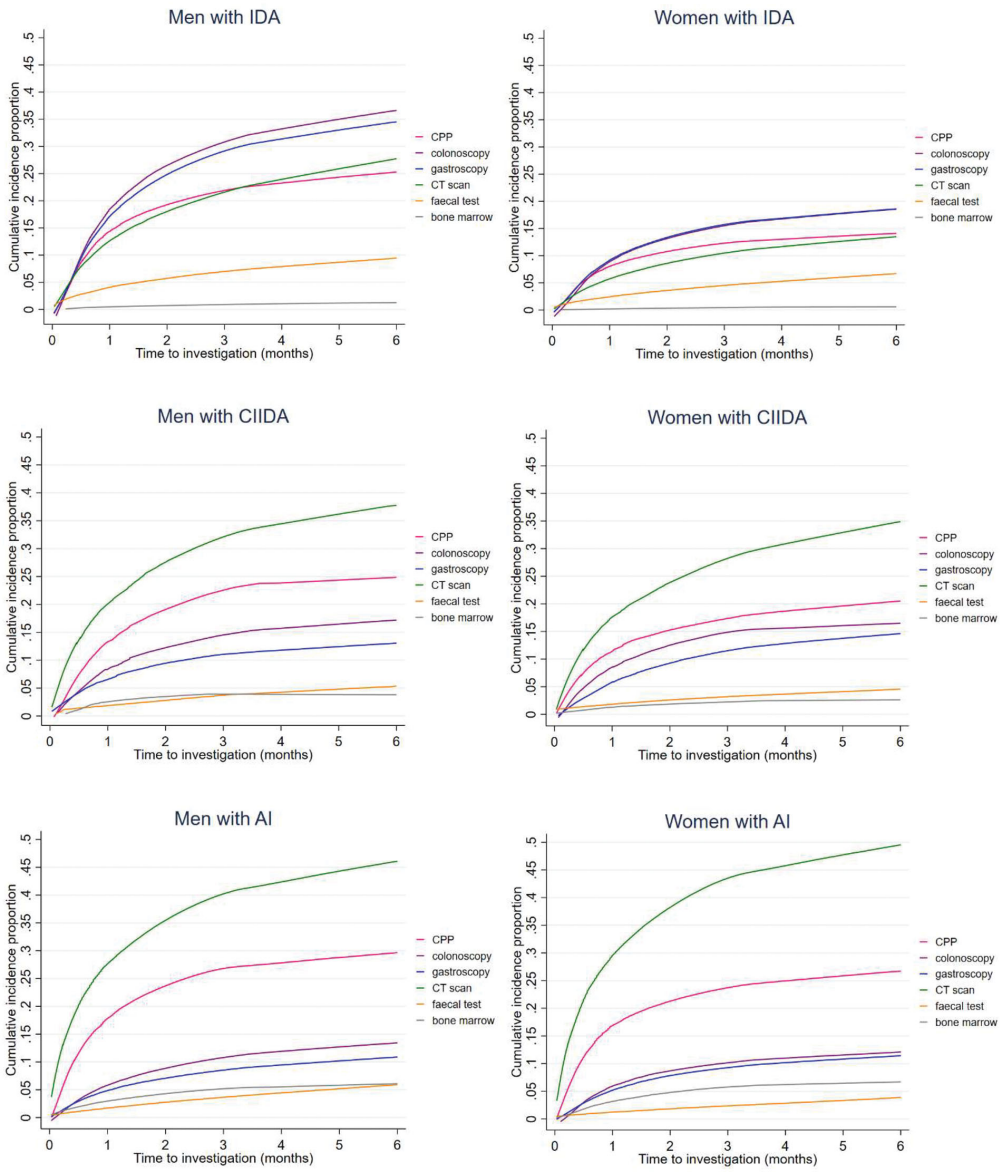

**Figure:**
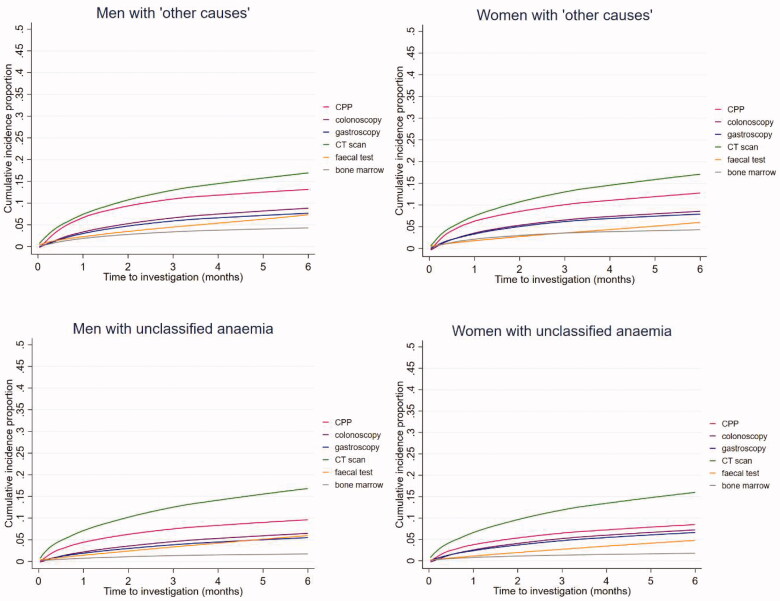

### Contacts to hospital and private practicing specialists

Among patients who had none of the diagnostic investigations, the majority had no contacts to hospital or to private practicing specialist (ranging from 87.4% (CI: 85.6–89.2) in patients with AI to 92.5% (CI: 91.9–93.1) in patients with unclassified anaemia).

### Associations between patient characteristics and diagnostic workup

Across all anaemia subtypes, patients aged 60–69 and 70–79 years were more likely to have any investigations performed compared to patients aged 40–49 years; the highest likelihood was seen in patients with IDA aged 70–79 years (HR: 3.28, CI: 2.91–3.69) (Table 2).

**Table 2.** Hazard ratios (HR) of having any of the included diagnostic investigations according to patient characteristics (stratified by anaemia subtypes) (*n* = 61,540).

Patient characteristics	IDA HR (95% CI)	CIIDA HR (95% CI)	AI HR (95% CI)	Other causes HR (95% CI)	Unclassified HR (95% CI)
Age groups, years					
40–49	1	1	1	1	1
50–59	1.91 (1.68–2.16)	1.41 (0.83–2.42)	1.40 (1.07–1.84)	1.57 (1.26–1.96)	1.86 (1.68–2.06)
60–69	3.24 (2.88–3.64)	1.78 (1.16–2.74)	1.60 (1.25–2.06)	1.80 (1.45–2.23)	2.40 (2.18–2.64)
70–79	3.28 (2.91–3.69)	2.14 (1.40–3.27)	1.55 (1.22–1.98)	1.68 (1.37–2.06)	2.07 (1.89–2.28)
80–89	2.08 (1.81–2.39)	1.09 (0.70–1.70)	1.01 (0.78–1.30)	1.13 (0.90–1.40)	1.35 (1.23–1.48)
Sex					
Men	1	1	1	1	1
Women	0.71 (0.65–0.78)	0.96 (0.76–1.23)	1.02 (0.92–1.13)	1.06 (0.95–1.18)	1.07 (1.02–1.12)
Educational level					
Low	1	1	1	1	1
Medium	1.03 (0.95–1.13)	0.87 (0.68–1.13)	1.05 (0.95–1.16)	1.18 (1.04–1.33)	1.10 (1.04–1.15)
High	1.00 (0.90–1.11)	0.94 (0.67–1.30)	0.95 (0.82–1.10)	1.13 (0.98–1.31)	1.06 (1.00–1.13)
Income					
Low	1	1	1	1	1
Medium	0.94 (0.86–1.04)	0.85 (0.65–1.11)	0.96 (0.86–1.08)	1.04 (0.92–1.17)	0.91 (0.87–0.96)
High	1.05 (0.95–1.17)	1.10 (0.81–1.48)	0.97 (0.85–1.11)	1.03 (0.89–1.19)	0.94 (0.89–1.00)
Civil status					
Living alone	1	1	1	1	1
Living with a partner	1.07 (0.99–1.16)	1.06 (0.85–1.34)	1.10 (0.99–1.21)	1.10 (0.99–1.22)	1.10 (1.05–1.14)
Anaemia severity^b^					
Mild	1	1	1	1	1
Moderate	2.04 (1.86–2.24)	1.96 (1.51–2.54)	1.66 (1.48–1.86)	2.41 (2.07–2.80)	2.42 (2.26–2.59)
Severe	4.62 (4.04–5.29)	3.92 (1.86–8.27)	2.60 (1.77–3.83)	4.02 (2.51–6.43)	7.39 (6.32–8.65)
No. of comorbidities					
0	1	1	1	1	1
1	0.98 (0.89–1.08)	0.60 (0.45–0.79)	0.85 (0.75–0.95)	1.01 (0.89–1.13)	0.95 (0.90–1.00)
2	1.02 (0.91–1.15)	0.57 (0.40–0.82)	0.75 (0.66–0.87)	0.92 (0.79–1.08)	0.89 (0.83–0.95)
≥3	0.89 (0.78–1.02)	0.62 (0.44–0.87)	0.65 (0.54–0.78)	0.91 (0.75–1.11)	0.84 (0.78–0.90)
Type of comorbidity^c^					
Cardiovascular disease	1.01 (0.92–1.11)	0.74 (0.57–0.96)	0.74 (0.66–0.83)	1.05 (0.94–1.18)	0.89 (0.84–0.94)
Hypertension	0.96 (0.87–1.05)	0.86 (0.65–1.14)	0.78 (0.69–0.88)	0.85 (0.74–0.98)	0.89 (0.84–0.94)
Mental illness	0.79 (0.68–0.92)	0.63 (0.41–0.95)	0.62 (0.50–0.77)	0.57 (0.45–0.72)	0.70 (0.64–0.76)
Diabetes	0.88 (0.78–1.00)	0.56 (0.37–0.85)	0.93 (0.78–1.11)	0.92 (0.77–1.11)	0.79 (0.74–0.85)
COPD	1.03 (0.88–1.20)	0.65 (0.45–0.93)	0.84 (0.70–1.00)	1.18 (0.96–1.46)	1.07 (0.99–1.17)
Neurological disorder	0.87 (0.67–1.14)	0.69 (0.34–1.37)	0.56 (0.39–0.81)	0.83 (0.61–1.13)	0.83 (0.73–0.95)
Arthritis	0.95 (0.61–1.46)	0.69 (0.22–2.18)	0.74 (0.49–1.13)	0.95 (0.55–1.65)	0.96 (0.79–1.17)
IBD	0.85 (0.57–1.26)	0.00 (0.00–0.00)	1.15 (0.68–1.96)	1.09 (0.69–1.72)	1.09 (0.89–1.34)
Liver disease	1.07 (0.77–1.50)	0.97 (0.52–1.81)	1.08 (0.80–1.45)	1.59 (1.12–2.25)	1.32 (1.12–1.57)
Kidney disease	1.29 (0.94–1.76)	1.05 (0.49–2.27)	0.87 (0.62–1.22)	0.79 (0.50–1.25)	0.83 (0.71–0.97)
Cancer	1.15 (0.99–1.34)	1.41 (0.99–2.00)	1.12 (0.96–1.30)	1.35 (1.12–1.62)	1.51 (1.41–1.62)

AI: anaemia of inflammation; CI: confidence intervals; CIIDA: combined inflammatory iron deficiency anaemia; COPD: chronic obstructive pulmonary disease; IBD: inflammatory bowel disease; IDA: iron deficiency anaemia; No.: number; Unclassified: the anaemia was not classifiable according to a guideline.

^a^Adjusted for age and sex.

^b^Anaemia severity was categorised into: mild anaemia (110 g/L to normal), moderate anaemia (80–110 g/L), severe anaemia (<80 g/L) (according to WHÓs guidelines). Units were converted from g/L to mmol/L (110 g/*L* = 6.8 mmol/L, 80 g/*L* = 5 mmol/L).

^c^Type of comorbidity was categorised according to the chronic disease groups.

Women with IDA were less likely to have any investigations performed compared to men with IDA (HR: 0.71, CI: 0.65–0.78), whereas women with unclassified anaemia were more likely to have any investigations performed compared to men with unclassified anaemia (HR: 1.07, CI: 1.02–1.12).

Across all anaemia subtypes, patients with severe anaemia were more likely to have any investigations performed compared to patients with mild anaemia (ranging from HR 2.60 (CI: 1.77–3.83) in patients with AI to HR 7.39 (CI: 6.32–8.65) in patients with unclassified anaemia).

Comorbidity was associated with a lower likelihood of having any investigations performed in patients with CIIDA, AI and unclassified anaemia compared to patients without comorbidity; the lowest HR was seen in patients with CIIDA and two comorbidities (HR: 0.57, CI: 0.40–0.82). Patients with mental illness were less likely to have any investigations performed compared to patients without mental illness (across all anaemia subtypes) (Table 2).

## Discussion

### Principal findings

This large-scale cohort study of nearly 60,000 patients revealed that around half of the patients with IDA, CIIDA, or AI had none of the cancer-relevant diagnostic investigations performed. Nearly eight in ten patients with unclassified anaemia had none of the diagnostic investigations performed. In patients having none of the diagnostic investigations, one to two in ten patients had other contacts to a hospital or private practicing specialist. Across all the anaemia subtypes, patients aged 60–79 years and patients with severe anaemia were more likely to have investigations performed. Comorbidity was associated with a decreased likelihood of investigations in patients with CIIDA, AI, and unclassified anaemia.

### Strengths and limitations

This study has several strengths. The high quality and completeness of the laboratory information systems and registries enabled us to establish a large population-based cohort with limited risk of selection bias and loss to follow up [17,20,28]. Moreover, the high validity of the registries and laboratory information systems, limited the risk of information bias due to missing and incorrect data [17,20,28]. The large study size made stratification possible and enhanced the statistical precision. The Danish citizens have free access to healthcare services, and the healthcare usage is known to be comparable across the regions [29]. This suggests that our findings are generalisable to other parts of Denmark and possibly to other countries with similar healthcare systems.

The anaemia guideline used in this study [5,7] is easily applicable in clinical settings and has previously been used in research [5,12]. The guideline includes IDA and AI, which are the two most common anaemia subtypes [4]. Moreover, it includes CIIDA as ferritin levels may be elevated due to inflammation [5].

An important limitation is the lack of information on patients’ symptoms. Furthermore, we had no knowledge of the indications for the investigations or contacts to hospitals and private practicing specialists. Likewise, the reasons for not investigating patients with new-onset anaemia are unknown, and there may be relevant reasons for not performing cancer-relevant investigations. In women <50 years of age presenting with IDA, menstrual bleeding is a dominant factor, which may be an explanation for no further investigations. Still, six in ten women with IDA aged 50 to 90 years had no cancer-relevant diagnostic investigations performed. Furthermore, clinicians may find it unnecessary to investigate mild anaemia. Still, three to six in ten patients with moderate or severe anaemia had no diagnostic investigations performed. However, all degrees of anaemia may represent underlying disease, including cancer. Further, around seven in ten anaemic patients diagnosed with cancer have mild anaemia.[30] As such, it is important to investigate all degrees of anaemia. Moreover, in frail elderly patients, the advantages and disadvantages of the diagnostic process of the suspected disease may be discussed. Further, we cannot rule out that a previous episode of anaemia could have occurred prior to the 15 months preceding the index date used as a rule-in period. Still, this would not have changed the conclusion of our study.

### Findings in relation to other studies

This is the first large-scale study investigating the diagnostic workup of possible cancer in patients with new-onset anaemia (detected in general practice) across different anaemia subtypes, including CIIDA and unclassified anaemia. The few existing studies investigating the diagnostic workup of patients with IDA [9,10,14,15] and AI [13] were small-scale studies [9,10,13–15], and some were done two decades ago [10,14,15].

In patients with IDA, we found a higher proportion of colonoscopies and gastroscopies compared to previous findings (24.9–36.9% vs. 6–21%) [9,10,14,15], except for one study reporting a comparable proportion of gastroscopies (27%) [15]. Our findings may reflect improved clinical practice, yet still not optimal approach. We found that patients aged 60–79 years, patients with severe anaemia, and men with IDA were more likely to receive diagnostic investigations, which was in line with previous findings [9,10,15].

In patients with AI, we reported a higher proportion of colonoscopies and gastroscopies compared to previous finding (11.0–13.3% vs. 1.9%) [13]. These fairly low proportions may reflect that other investigations are more recommendable in these patients (e.g. NSSC-CPP including CT scans) [11]. Further, we reported a higher proportion of CT scans compared to previous finding (44.4–47.3% vs. 4.5%) [13]. However, the previous finding may reflect a different clinical practice as patients referred to a medical specialist (54%) had a CT scan included as standard [13].

It can be a clinical challenge to differentiate potentially underlying malignancy from pre-existing comorbidities in patients with AI. However, notably, patients with mental illness were less likely to receive investigations across all anaemia subtypes. This is in line with previous findings that patients with mental illness are less likely to receive investigations for co-existing comorbidities [30].

In the present study, a large proportion of patients had unclassified anaemia, and no diagnostic investigations were performed in the majority of these patients. As we could not identify any studies reporting on the implications of having unclassified anaemia, the implications remain unknown.

### Meaning of the study

Anaemia is associated with increased morbidity and mortality [1–3]. Searching for the underlying cause of anaemia is essential for timely diagnosis of potentially underlying disease, including undiagnosed malignancy [2,6,16]. Our results indicate that around half of the patients with new-onset ‘iron deficiency anaemia’, ‘anaemia of inflammation’, or ‘combined inflammatory iron deficiency anaemia’ in general practice had no cancer-relevant diagnostic procedures performed. In some cases, this may represent missed diagnostic opportunities.

Future interventions are required to improve the diagnostic workup of possible cancer in patients with new-onset anaemia, for example, alert systems linked to abnormal laboratory results and clinical decision support. Additionally, future research is needed to determine the clinical outcomes in patients with different anaemia subtypes, including the implications of having unclassified anaemia.
